# Cerebrospinal Fluid Bacillary Load by Xpert MTB/RIF Ultra Polymerase Chain Reaction Cycle Threshold Value Predicts 2-Week Mortality in Human Immunodeficiency Virus–Associated Tuberculous Meningitis

**DOI:** 10.1093/cid/ciaa1444

**Published:** 2020-09-28

**Authors:** Emily M Martyn, Ananta S Bangdiwala, Enock Kagimu, Morris K Rutakingirwa, John Kasibante, Michael Okirwoth, Gavin Stead, Vincent Wadda, Matthew F Pullen, Tyler D Bold, David B Meya, David R Boulware, Nathan C Bahr, Fiona V Cresswell

**Affiliations:** 1 Infectious Diseases Institute, Kampala, Uganda; 2 University of Minnesota, Minneapolis, Minneapolis, USA; 3 Mulago National Referral Hospital, Kampala, Uganda; 4 Department of Medicine, College of Health Sciences, Makerere University, Kampala, Uganda; 5 University of Kansas Medical Center, Kansas City, Kansas, USA; 6 Clinical Research Department, London School of Hygiene and Tropical Medicine, London, United Kingdom

**Keywords:** tuberculous meningitis, TB meningitis, GeneXpert Ultra, ultra

## Abstract

**Background:**

The World Health Organization recommends GeneXpert MTB/RIF Ultra (Xpert Ultra), a fully automated polymerase chain reaction (PCR) assay, as the initial tuberculous meningitis (TBM) diagnostic test. The assay’s PCR cycle threshold (Ct) values represent the number of PCR cycles required for probe signal to be detected (low Ct value = high bacillary load) and may approximate tuberculosis (TB) bacillary load. We measured the relationship between cerebrospinal fluid (CSF) TB bacillary load with mortality.

**Methods:**

We prospectively enrolled 102 human immunodeficiency virus (HIV)–positive Ugandans with probable or definite TBM from April 2015 to August 2019. Xpert Ultra Ct tertiles and semi-quantitative categories were separately analyzed as predictors of 2-week mortality. We investigated associations between Ct and baseline clinical and CSF parameters.

**Results:**

Subjects with Ct values in the low tertile (ie, high bacillary load) had 57% 2-week mortality—worse than the intermediate (17%) and high (25%) Ct tertiles and Xpert Ultra–negative (30%) probable TBM cases (*P* = .01). In contrast, the reported semi-quantitative Xpert Ultra categorization was less precise; with the medium to low category trending toward worse 2-week survival (42%) compared with very low (28%), trace (26%), and negative (30%) categories (*P* = .48). Ct tertile was significantly associated with baseline CSF lactate (*P* = .03).

**Conclusions:**

High CSF TB bacillary load, as measured by Xpert Ultra Ct tertile, is associated with an almost 2-fold higher 2-week mortality in HIV-associated TBM and is a better predictor than the reported Xpert Ultra semi-quantitative category. Xpert Ultra Ct values could identify TBM patients at increased risk of death who may benefit from enhanced supportive care.

## Teaser

High cerebrospinal fluid (CSF) tuberculosis bacillary load, as measured by GeneXpert Ultra cycle threshold tertile, is associated with an almost 2-fold higher 2-week mortality in HIV-associated tuberculosis meningitis and may be a better predictor of outcome than the reported Xpert Ultra semi-quantitative category.

Tuberculosis (TB) accounts for approximately 1.3 million deaths from 10 million new cases annually worldwide, with the highest burden in sub-Saharan Africa [1]. Tuberculous meningitis (TBM) occurs in approximately 1%–5% of global TB cases and is the second leading cause of meningitis in many African settings [2–5]. TBM has a devastating impact, with approximately 40% in-hospital mortality and up to 60% of survivors left with neurological disability [6, 7].

TBM is notoriously challenging to diagnose, often relying upon clinical suspicion, which may contribute to high mortality. Historically, cerebrospinal fluid (CSF) culture has been the mainstay of confirmatory testing, which takes at least 2 weeks to provide results, often too late to impact clinical management [8]. The development of the Xpert MTB/RIF assay (Xpert) was a major step forward in TBM diagnosis. This fully automated, cartridge-based polymerase chain reaction (PCR) assay provides a semi-quantitative result within 2 hours. The newer Xpert MTB/RIF Ultra (Xpert Ultra, Cepheid, Sunnyvale, California), a reengineered version of Xpert using the same platform, was recommended by the World Health Organization in 2017 [9]. Ultra has improved sensitivity compared to the original Xpert; a recent study in a cohort of Ugandan human immunodeficiency virus (HIV)–positive adults with TBM demonstrated a 76.5% sensitivity for probable/definite TBM compared to 45% and 43% for Xpert and culture, respectively [8, 10].

The reason for Xpert Ultra’s improved sensitivity is its lower limit of detection of 16 colony-forming units per milliliter (CFUs/mL), vs 114 CFUs/mL for Xpert [11]. This is achieved primarily by incorporating a larger PCR reaction chamber and adding 2 multicopy *Mycobacterium tuberculosis* DNA amplification targets (*IS6110* and *IS1081*), in addition to the 4 probes directed at mutations in the rifampicin resistance–determining region of the *rpoB* gene (*rpoB1–4)* [9]. The PCR cycle threshold (Ct) values represent the number of PCR cycles required for each probe signal to reach a detection threshold and may approximate TB bacillary load. When DNA amplification reaches the detection threshold at any of the targets, the result “MTB detected” is given, and depending on the Ct values and configuration of probe positivity, 5 semi-quantitative categories of TB bacillary load are reported: trace, very low, low, medium, and high [9]. A nuance of the Ultra assay is that a “trace” category result occurs when *rpoB* probes are negative and *IS6110/IS8110* probes are positive, regardless of the Ct value. This nuance may make Ct value a better correlate of bacillary load than the semi-quantitative category.

Various methods have been used to estimate TB bacillary load, including time to culture positivity, Ziehl-Neelsen smear microscopy, and *M. tuberculosis* molecular bacterial load assay [12]. One study found that time to CSF culture positivity is independently associated with mortality in HIV-negative patients (hazard ratio, 1.37 [95% confidence interval, 1.02–1.84]), suggesting that an increased bacterial load may be associated with worse outcomes [13]. However, these methods are time consuming, are difficult to apply to TBM due to paucibacillary CSF, and are not widely available in low- and middle-income countries; thus, their role in TBM is uncertain. Xpert and Xpert Ultra provide new opportunities to investigate the relationship of bacillary burden and outcome in TBM. We have recently shown that the Xpert Ultra semi-quantitative category correlates with days to CSF culture positivity [8]. In addition, a recent study found that high CSF TB bacillary load, as measured by Xpert Ct value, was associated with increased disease severity, CSF neutrophil count, and CSF cytokine concentrations and predicted new neurological events after starting treatment, but not death [14]. With the introduction of the new, more sensitive Xpert Ultra, and in a population of predominantly HIV-positive persons in Uganda with TBM, we examined whether CSF TB bacillary load was associated with clinical outcome and CSF biochemical or cellular parameters.

## MATERIALS AND METHODS

### Study Setting and Population

We prospectively evaluated adults aged ≥18 years suspected to have meningitis presenting to Mulago National Referral Hospital and Mbarara Regional Referral Hospital in Uganda between April 2015 and August 2019 as part of the screening process for 2 clinical trials (XXX [ASTRO-CM]: NCT01802385 and High-Dose Intravenous and Oral Rifampicin to ImproveSurvival of Adult Tuberculous Meningitis [RifT]: ISRCTN42218549). The parent trials received relevant institutional review board and regulatory approvals, and all participants or surrogates (in the case of altered mental status) gave informed consent for lumbar puncture and storage of samples for future research purposes.

### Uniform Clinical Case Definition

Subjects with probable or definite TBM as defined by the consensus uniform clinical case definition were included. Definite TBM includes any CSF positivity by microscopy, culture, or PCR. Probable TBM cases have negative CSF TB microbiology but reach a score of >12 points or >10 points with or without brain imaging, respectively, based on clinical, radiological, and CSF criteria and evidence of confirmed TB in another anatomical location [15].

### CSF Testing

CSF samples from diagnostic lumbar punctures were tested at the bedside for cryptococcal antigen (CrAg lateral flow assay; IMMY, Norman, Oklahoma), glucose (OneTouch Select glucose meter, Lifescan, Inverness, United Kingdom), and lactate (Lactate Plus Meter, Nova Biomedical, Waltham, Massachusetts). Approximately 1 mL of CSF was sent for routine testing (cell count and differential, protein, Gram stain, and culture). Patients without cryptococcal meningitis (CSF CrAg negative) were evaluated for TBM. With regard to TBM testing, from February 2015 to December 2018 the remaining sample was centrifuged at 3000*g* for 20 minutes. Surplus supernatant was removed and the cell pellet was resuspended in 2 mL of supernatant and divided between Xpert, Xpert Ultra, and mycobacterial growth inhibitor tube (BacTec 960, Becton Dickinson, Franklin Lakes, New Jersey). For samples collected in 2019, an uncentrifuged 2-mL CSF sample was tested with Xpert Ultra.

### Study Treatment and Follow-up

Following TBM diagnosis, all patients were treated with rifampicin, isoniazid, pyrazinamide, ethambutol, and adjunctive corticosteroids (dexamethasone 0.4 mg/kg/day in week 1, 0.3 mg/kg/day in week 2, then tapered over 2 months). During the majority of our diagnostic study period, the follow-up duration was 2 weeks. Research medical officers recorded a clinical assessment and baseline demographics on day 1, and vital status at 2 weeks. From 14 January 2019, eligible participants were enrolled into the RifT trial, a phase 2 pharmacokinetic study investigating high-dose rifampicin. For the purposes of this analysis, follow-up was limited to 2 weeks, since only a minority of participants had longer-term follow-up after January 2019.

### Statistical Analysis

We used Ct values from the multicopy amplification targets *IS6110*/*IS1081* (unique to Xpert Ultra) as a surrogate for TB bacillary load. A positive Xpert Ultra result always yields a Ct value for the *IS6110*/*IS1081* targets; hence, these were used for analysis rather than the *rpoB* targets, which were negative in 27 of 71 positive Xpert Ultra results. A high Ct value signifies an increased number of PCR amplification cycles required for TB detection, and therefore may correlate with a low bacillary load. Ct values were categorized into tertiles: low (16.2–20.0), intermediate (20.1–25.0), high (25.1–32.7), and negative. Participants with a negative value were assigned an artificially high Ct value of 40 for analysis purposes. Baseline characteristics were compared across all categories with Kruskal-Wallis tests for continuous variables and χ ^2^ tests for categorical variables. Two-week survival was plotted via Kaplan-Meier curves for Ct tertiles and negative Xpert Ultra and compared via a log-rank test. A second Kaplan-Meier plot was calculated for semi-quantitative values (trace, very low, medium-low, and negative). Medium and low were grouped together due to small number of participants in those categories. All statistical analysis was done with SAS version 9.4 software (SAS Institute, Cary, North Carolina).

## RESULTS

CSF samples from 102 adults with TBM were tested with Xpert Ultra. One participant was excluded due to a missing semi-quantitative category result; of the remaining 101 participants, 70 (69%) had a positive Xpert Ultra and 31 (31%) had a negative result. The semi-quantitative category results were as follows: 0 high, 5 medium, 20 low, 18 very low, and 27 trace. Of the 31 participants with a negative Xpert Ultra, 5 had a positive CSF culture and 1 had a positive acid-fast smear. The median age of participants was 32 (interquartile range [IQR], 29–38) years, and 40% were women. The study population were 95% HIV-positive with a median CD4 of 78 (IQR 37–131) cells/μL, and 50% of participants were receiving antiretroviral therapy (ART) at presentation for a median duration of 4.4 months. The British Medical Research Council (BMRC) TBM grade was grade 2 or 3 for 91% of participants at enrollment. Baseline demographics did not significantly differ between Ct tertiles (Table 1).

**Table 1.** Comparison of Baseline Demographics, Clinical Features, and Cerebrospinal Fluid Biochemical and Cellular Parameters According to GeneXpert MTB/RIF Ultra Cycle Threshold Tertile Categories

Characteristic	Low Tertile (Ct 16.2–20.0) (n = 22)	Intermediate Tertile (Ct 20.1–25.0) (n = 24)	High Tertile (Ct 25.1–32.7) (n = 24)	*P* Value^a^
Baseline demographics				
Age, y	32 (29–36)	34 (32–38)	31 (28–37)	.26
Sex, female	10 (46%)	10 (42%)	7 (29%)	.49
HIV details				
HIV positive	20 (91%)	23 (96%)	24 (100%)	.31
Currently on ART	10 (48%)	12 (52%)	13 (54%)	.91
Months on ART^b,c^	9.7 (3.1–22.2)	2.3 (0.3–7.0)	1.1 (0.7–3.4)	.18
CD4 count^c^, cells/μL	58 (48–73)	89 (56–210)	59 (14–117)	.36
Clinical details				
Focal neurological deficit	5 (23%)	4 (17%)	10 (42%)	.13
Fever	18 (82%)	19 (79%)	20 (83%)	.65
BMRC TBM grade				
1	2 (10%)	5 (21%)	2 (9%)	.46
2	13 (62%)	16 (67%)	14 (61%)	
3	6 (29%)	3 (13%)	7 (30%)	
Baseline CSF characteristics				
Total WBC count^c^, cells/μL	80 (4–245)	35 (4–250)	35 (4–165)	.50
% lymphocytes	95 (65–100)	85 (69–100)	93 (65–100)	.92
Opening pressure^c^, mm H_2_O	190 (100–270)	230 (100–290)	205 (130–300)	.97
Protein^c^, mg/dL	118 (46–208)	156 (90–316)	144 (101–200)	.58
Lactate^c^, mmol/L	10.6 (9.5–12.0)	7.6 (6.9–9.5)	7.4 (4.6–9.8)	.03
Glucose^c^, mg/dL	27 (18–33)	32 (18–70)	30 (21–60)	.44

Data are presented as median (interquartile range) or no. (%).

Abbreviations: ART, antiretroviral therapy; BMRC, British Medical Research Council; CSF, cerebrospinal fluid; Ct, Xpert Ultra polymerase chain reaction cycle threshold; HIV, human immunodeficiency virus; TBM, tuberculous meningitis; WBC, white blood cell.

^a^Kruskal-Wallis for medians; χ ^2^ test for proportions.

^b^Among those on ART at diagnosis.

^c^Incomplete data: months on ART (n = 29); CD4 cell count (n = 41); total WBC count (n = 65); opening pressure (n = 39); CSF protein (n = 60); CSF lactate (n = 31); CSF glucose (n = 54).

**Figure 1.**
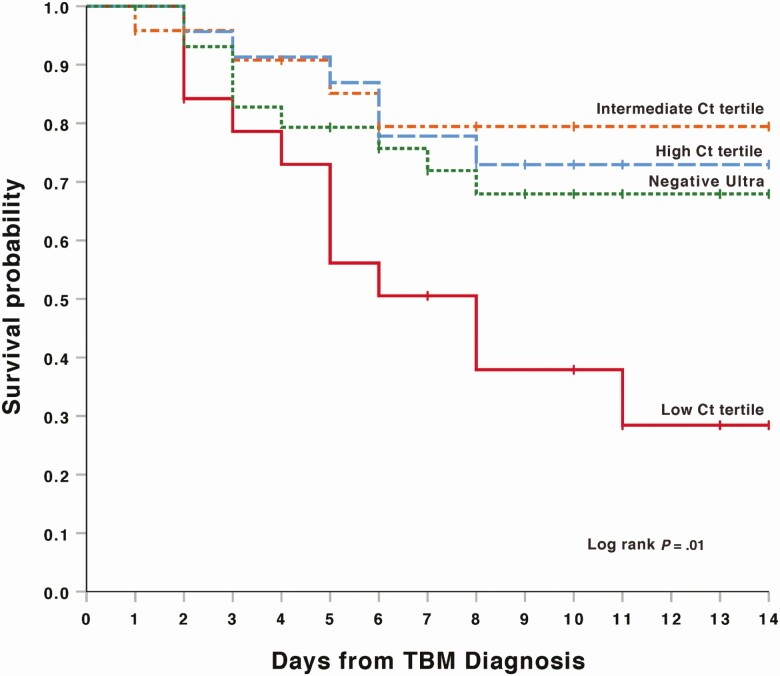
Two-week survival by Xpert MTB/RIF Ultra polymerase chain reaction cycle threshold (Ct) level among participants with probable or definite tuberculous meningitis (TBM) in Kaplan-Meier model. Mortality by subgroup was as follows: low Ct tertile, 57% (12/21); intermediate Ct tertile, 17% (4/24); high Ct tertile, 25% (6/24); and negative Ultra, 30% (9/30).

Ninety-nine participants were included in the survival analysis, since 2 participants were missing outcome data. Participants with a CSF Xpert Ultra Ct value result in the low tertile (ie, high bacillary load) had a 57.1% 2-week mortality, significantly worse than other categories (log-rank *P* = .01; Figure 1). The lowest 2-week mortality was found within the intermediate Ct tertile group (16.7%). Participants within the high Ct tertile (low bacillary load) had a lower 2-week mortality than the negative Xpert Ultra group (25.0% and 30.0%, respectively).

**Figure 2.**
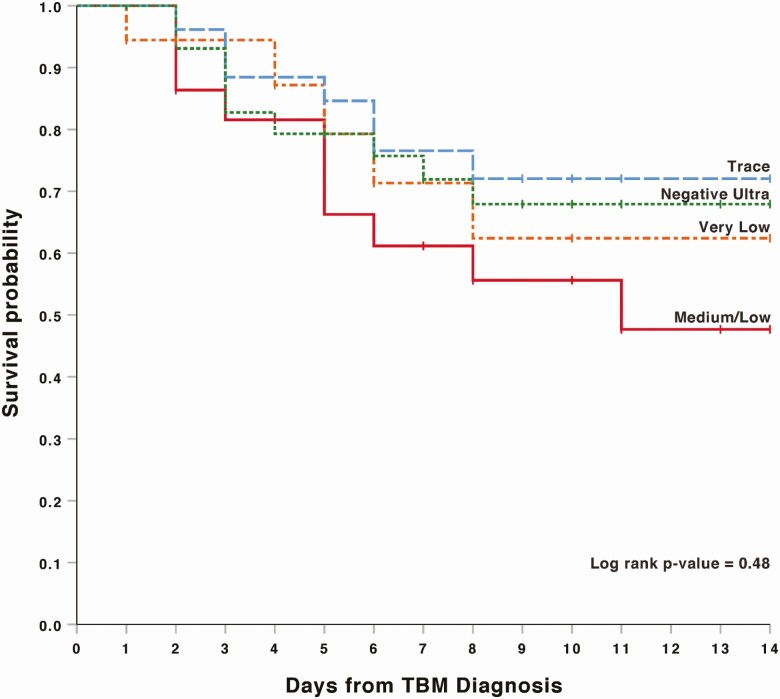
Two-week survival by Xpert MTB/RIF Ultra semi-quantitative value among participants with probable or definite tuberculous meningitis (TBM) in Kaplan-Meier model. Mortality by subgroup was as follows: medium/low, 42% (10/24); very low, 28% (5/18); trace, 26% (7/27); and negative Ultra, 30% (9/30).

In the survival analysis based on Xpert Ultra semi-quantitative categories, participants in the medium and low groups (combined) trended toward higher 2-week mortality (42%) compared with very low (28%), trace (26%), and negative (30%) persons (log-rank *P* = .48; Figure 2). We replotted Kaplan-Meier survival curves for both Ct tertiles and semi-quantitative values in a sensitivity analysis excluding the 6 persons with negative CSF Xpert Ultra, but with a positive culture or smear result. For both Ct tertiles and semi-quantitative groups, the mortality remained the same, except in the Ultra negative group where mortality decreased from 30% to 25% in both analyses. The log-rank *P* values remained relatively unchanged for both Ct tertiles and semi-quantitative groups (*P* = .01 and .41, respectively).

The Ct tertile was not associated with HIV status (*P* = .31), baseline CD4 cell count (*P* = .36), or the percentage of participants on ART (*P* = .91). Participants in the low Ct tertile group (high bacillary load) had a median ART duration of 9.7 months, whereas participants in the intermediate and high Ct tertile groups trended toward a shorter median duration of ART therapy (2.3 and 1.1 months, respectively), though this difference was not statistically significant (*P* = .18). There was no association with clinical features such as fever (*P* = .65), BMRC TBM severity grade (*P* = .46), or focal neurologic deficit (*P* = .13). With respect to baseline CSF parameters, there was a significant difference observed between Ct tertiles in CSF lactate, with the highest lactate observed in the low Ct tertile (high bacillary load) (*P* = .03). There were no differences between Ct tertiles in opening pressure (*P* = .97), protein (*P* = .58), total white blood cell (WBC) count (*P* = .50), or glucose (*P* = .44) (Table 1).

## Discussion

In our study of HIV-associated TBM, low Ct tertile value (high CSF TB bacillary load), as measured by Xpert Ultra, was associated with an almost 2-fold increased mortality compared to those with intermediate and high Ct tertiles. An intermediate CSF Ct tertile value was associated with the most favorable 2-week outcome. Interestingly, association of 2-week mortality was statistically significant for Ct tertiles but not for semi-quantitative categories. There was a significant difference in CSF lactate between Ct tertiles.

In 2019, Thuong et al investigated the association between pretreatment CSF TB bacillary load by Xpert Ct value and TBM outcome in 692 Vietnamese adults [14]. This study found that higher bacterial loads were associated with increased disease severity (TBM grade 1 vs 3: odds ratio, 1.59; *P* = .001), increased CSF neutrophil count (*r* = .364; *P* < .0001), and 10 measured cytokine concentrations (*r* = .438; *P* < .0001) [14]. In addition, high TB bacillary load predicted new neurological events after treatment initiation but not death. In contrast, our data suggested an association between bacillary load and 2-week mortality but did not find an association with disease severity (by BMRC TBM severity grade) or focal neurological deficit. We also did not observe an association between CSF Ct tertile and CSF inflammation. However, potential comparison between studies is limited by measurement of different surrogate markers of inflammation (CSF protein and WBC count in Uganda vs CSF neutrophil and cytokine concentrations in Vietnam), differences in HIV prevalence between populations (which can affect the cellularity of CSF and neutrophil count in HIV-associated TBM), and our smaller sample size, making marginal associations more difficult to measure [16–18]. Further divergent results may be due to the use of Xpert Ultra, rather than the original Xpert. It is possible that we were able to draw distinctions in mortality because we could detect more cases with lower CSF bacillary load. In addition, our cohort of mainly HIV-positive Ugandans presenting with severe disease was markedly different to the Vietnamese study, in which 58% were HIV negative and 38% presented with TBM grade 1. This distinct cohort may have different host responses and CSF bacillary loads. Another important geographic consideration is variation in the copy number of *IS6110* and *IS1081* sequences present within local *M. tuberculosis* strains, which supports further validation of this work in different populations before it is considered as a prognostic biomarker.

Our observation that Ct tertiles, rather than semi-quantitative values, are a better predictor of mortality could be explained by the nature of the in-built semi-quantitative categorization process. The semi-quantitative Xpert Ultra categories are determined by the configuration of positivity of 4 *rpoB* probes and multicopy amplification targets *IS6110/IS1081.* A “trace” category occurs if the *rpoB* probes are negative, regardless of the *IS* Ct values [9]. Data from the current study suggest that looking at Xpert Ultra *IS6110/IS1081* Ct values may be a better predictor of mortality compared with the semi-quantitative category.

We report that participants with an intermediate Ct tertile had the most favorable 2-week outcome, followed by those with low CSF TB burden (high Ct tertile and negative Xpert Ultra). The worst outcome occurred in patients within the low Ct tertile group (high bacillary load). It is noteworthy that those with lowest CSF bacillary burden appear to have worse outcomes than those with an intermediate bacillary load. It is possible that this difference may change with a larger sample size. However, this may also represent an excessive immune response that successfully curtails bacterial replication but leaves a trail of immunopathology in its wake. Studies suggest that adverse outcomes are associated with both an excessive and inadequate immune response in TBM, and the same is true in cryptococcal meningitis where the damage-response parabola has been well described [19]. For example, HIV-negative Vietnamese adults with TBM had an increased mortality when they possessed a single-nucleotide polymorphism in the *LTA4H* rs17525485 allele, resulting in either a hyper- or hypo-inflammatory state [16]. Worse outcomes have also been associated both with high CSF neutrophils in an Indonesian, predominantly HIV-negative cohort, and conversely with acellular CSF in adults with HIV-associated TBM in Uganda [13, 18]. Therefore, it is conceivable that mortality differences observed in our study reflect the varying host immune response, which either curtails bacillary replication through an excessive inflammatory response with ensuing tissue damage or allows unchecked TB replication in the CSF due to a paucity of inflammatory response. CSF cytokine and other studies of host response would be helpful in further understanding these findings.

We observed a significant difference in CSF lactate across Ct tertiles, with the highest CSF lactate observed in the low Ct tertile group (ie, highest bacillary load). Previous studies suggest that lactate levels between 5 and 10 mmol/L support a diagnosis of TBM and high initial lactate levels are associated with death [20]. Further investigation is required into the utility of CSF lactate as a potential diagnostic and prognostic biomarker in TBM. Our data also reveal a nonsignificant trend toward shorter duration of ART in participants within the intermediate and high Ct tertile groups (intermediate and low bacillary loads). This difference may be explained by unmasking immune reconstitution inflammatory syndrome (IRIS), although formal IRIS case adjudication was not performed. Further work to carefully characterize unmasking TBM-IRIS with correlations to Xpert Ultra results is required.

A limitation of this study is that 17 of 101 participants received high-dose rifampicin as part of a clinical trial. However, these participants were distributed evenly across Ct tertile groups (*P* = .93). In addition, there is currently no definitive evidence that high-dose rifampicin impacts survival, and final analysis of the parent study did not identify differences in mortality between arms (unpublished data) [21, 22]. Another limitation is the relatively small cohort size, which may have reduced the power to detect secondary associations. Furthermore, we used Ct value as a surrogate for bacillary load (in the absence of an effective bacillary load assay), but this may be an imperfect proxy. Our analysis was limited to 2-week follow-up, which is justified by the significant early mortality in TBM described in the literature [13, 23]. Whether this association with acute 2-week mortality translates into ongoing differential mortality in the longer term should be studied in future observational cohorts. In addition, further work including correlation of Ct tertiles with clinical descriptions of IRIS and immunological studies, such as cytokine studies or transcriptomics, may help further explain our findings. As this is a first report of Xpert Ultra Ct values being used as a predictor of outcomes in TBM, our observation requires independent confirmation in non-HIV populations, in other geographical settings, and in larger cohorts.

In conclusion, we report that a low Xpert Ultra Ct tertile (high CSF TB bacillary load) is associated with increased mortality in HIV-associated TBM and that Ct tertiles are more accurate in predicting early TBM-related mortality than Xpert Ultra semi-quantitative categories alone. This study raises the possibility of Xpert Ultra Ct values being used to identify patients at greatest risk of death and who may benefit from intensified treatment or enhanced supportive care.

## Notes

***Acknowledgments.*** The authors are appreciative of the study team and hospital staff working continuously to provide compassionate care to the participants and patients at Mulago National Referral Hospital.

***Financial support.*** This work was supported by the National Institute of Neurological Disorders and Stroke (grant numbers R01NS086312 and K23NS110470); the Fogarty International Center; and the National Institute of Allergy and Infectious Diseases (grant numbers T32AI055433 and R01AI145437). F. V. C. is supported through a Wellcome Trust Clinical PhD Fellowship (grant number 210772/Z/18/Z).

***Potential conflicts of interest.*** The authors: No reported conflicts of interest. All authors have submitted the ICMJE Form for Disclosure of Potential Conflicts of Interest.
